# Impact of preoperative statin medication on long-term outcomes after pancreatoduodenectomy for ductal adenocarcinoma: an international multicentric cross-sectional study

**DOI:** 10.1007/s00432-023-05426-2

**Published:** 2023-09-23

**Authors:** Gaëtan-Romain Joliat, Sérgio Gaspar-Figueiredo, Ismail Labgaa, Dionisios Vrochides, Julie Perinel, Mustapha Adham, Nicolas Demartines, Markus Schäfer

**Affiliations:** 1 Department of Visceral Surgery, Lausanne University Hospital CHUV, University of Lausanne (UNIL), Rue du Bugnon 46, 1011 Lausanne, Switzerland; 2 Graduate School of Health Sciences, University of Bern, Bern, Switzerland; 3 Division of Hepatobiliary and Pancreatic Surgery, Department of Surgery, Carolinas Medical Center, Charlotte, USA; 4 Department of Surgery, Edouard Herriot Hospital, Lyon, France

**Keywords:** Pancreas surgery, Outcomes, Medication, Prognosis

## Abstract

**Purpose:**

Statin treatment has been shown in certain population studies and meta-analyses to improve survival of patients with pancreatic ductal adenocarcinoma (PDAC). This study assessed if patients with statin treatment had better overall survival (OS) and disease-free survival (DFS) after upfront pancreatoduodenectomy for PDAC.

**Methods:**

Consecutive PDAC patients were retrospectively collected from three centers in Europe and USA (study period: 2000–2017). Adult patients who underwent upfront pancreatoduodenectomy and survived the first 90 postoperative days were included. Patients with metastasis at diagnosis or with macroscopic incomplete resection were excluded. Patients were considered under statin if started at least one month before pancreatoduodenectomy. Survival rates were calculated using Kaplan–Meier method and compared with log-rank test.

**Results:**

A total of 496 patients were included. Median age was 67 years (IQR 59–75), 48% (n = 236) were women, and 141 patients (28%) received statin treatment already preoperatively. Patients with and without statin treatment were comparable in terms of demographics and pre-/intraoperative characteristics, except for age and pre-existing diabetes. Median OS and DFS were similar in patients with and without statin treatment (OS: 29, 95% CI 22–36 vs. 27 months, 95% CI 22–32, p = 0.370, DFS: 18, 95% CI 14–22 vs*.* 16 months, 95% CI 14–18, p = 0.430). On multivariable Cox regression, lymph node involvement (HR 1.9, 95% CI 1.6–2.2, p < 0.001), tumor differentiation (HR 1.3, 95% CI 1.1–1.6, p = 0.003), and postoperative chemotherapy (HR 0.5, 95% CI 0.4–0.7, p < 0.001) were predictors of OS, whereas statin treatment was not a prognostic factor (HR 0.9, 95% CI 0.7–1.2, p = 0.376).

**Conclusion:**

In this international cohort of PDAC patients, statin treatment did not influence survival after upfront pancreatoduodenectomy. Nodal involvement, tumor differentiation, and postoperative chemotherapy were independent predictors of OS.

## Introduction

Pancreatic ductal adenocarcinoma (PDAC) is the most common form of pancreatic cancer, one of the most lethal cancers with a 5-year survival rate of less than 10% (Fristrup et al. 2016). Radical surgical resection represents the backbone of any curative approach. However, surgical interventions, such as pancreatoduodenectomy (PD) are complex and its associated postoperative morbidity is still high. Despite recent advances in surgical techniques and perioperative care, the long-term outcomes for patients with pancreatic cancer remain dismal (Conroy et al. 2018). There is a need to find novel approaches that could improve survival rates by reducing early cancer recurrence.

Statins are nowadays widely used drugs to lower serum cholesterol levels and to reduce the risk of cardiovascular disease by a direct inhibition of the enzyme HMG (hydroxymethylglutaryl)-CoA-reductase. Recently, there has been growing interest in the potential anti-tumor effects of statins (Yuan et al. 2022). Several preclinical and clinical studies have suggested that statins may inhibit the growth and dissemination of pancreatic cancer cells (Tamburrino et al. 2020). However, the impact of statin use on long-term outcomes in patients undergoing PD for pancreatic cancer is not well established.

The aim of this cross-sectional study was to evaluate whether preoperative statin use is associated with improved long-term survival and reduced cancer recurrence in patients undergoing upfront PD for ductal adenocarcinoma of the pancreatic head.

## Methods

### Patients

Inclusion criteria were patients > 18 years old and who underwent upfront PD for ductal adenocarcinoma proven on histopathology from January 2000 to December 2017. Exclusion criteria were absence of consent to research, macroscopic incomplete resection (R2 resection), perioperative death within 90 postoperative days, or metastasis at time of diagnosis. Patients with neoadjuvant treatment were not considered.

Patients were issued from 3 international tertiary and referral centers: Edouard-Herriot Hospital (Lyon, France), Carolinas Medical Center (Charlotte, USA), and Lausanne University Hospital CHUV (Lausanne, Switzerland). Postoperative complications were defined according to the Clavien classification (Dindo et al. 2004), and the Comprehensive Complication Index (CCI) was calculated (Slankamenac et al. 2013). Delayed gastric emptying, pancreatic fistula, and hemorrhage were based on the International Study Group for Pancreatic Surgery definitions (Bassi et al. 2005; Wente et al. 2007a, b). Surgical-site infection was defined according to the Centers for Disease Control and Prevention (Horan et al. 1992).

### Objectives and endpoints

The main objective was to compare the survival rates of patients with versus without statin treatment. The primary endpoint was the overall survival in months (OS).

Secondary objectives were to evaluate if patients with statin treatment presented less recurrence. Secondary endpoints were disease-free survival (DFS) and recurrence rate.

### Statin treatment

Patients were considered under a preoperative statin treatment if the statin intake was started at least one month before PD. Only statin treatments were considered. Other anti-lipid drugs such as fibrates were not taken into account. All subclasses of statin were included.

### Statistics

Continuous data were summarized using median and interquartile range (IQR), and categorical data were summarized with number and percentage. Mann–Whitney U tests were used to compare parametric continuous data, while chi-square tests were performed for categorical data. Survival curves (OS and DFS) were calculated using Kaplan-Maier method and compared with the log-rank test. Median follow-up was calculated using the inverse Kaplan-Maier method. Predictive factors of survival were calculated using uni- and multivariable Cox regression analyses. Continuous variables were dichotomized for the Cox regression analyses: age (> 60 vs. ≤ 60), body-mass index (> 25 kg/m^2^ vs. ≤ 25 kg/m^2^), American Society of Anesthesiologists score (I/II vs. III/IV), tumor size (T1-2 vs*.* T3-4), and CA19-9 (> 42 U/ml vs. ≤ 42 U/ml)(Shimagaki et al. 2023). Only factors with p < 0.1 on univariable analysis were included in the multivariable analysis. A two-tailed p-value < 0.05 was considered significant. SPSS© 28.0 Statistics for Mac was used for all statistical analyses (IBM Corp., Armonk, NY, USA).

## Results

### Patients

A total of 496 patients were included with a median age of 67 years (IQR 59–75). Forty-eight percent of the patients (236/496) were women. The overall morbidity rate was 64% (319/496) and the median CCI was 8.7 (IQR 0–27.6). Median OS and DFS were 27 months (95% CI 23–31) and 17 months (95% CI 15–19), respectively.

There were 141 patients (28%) who had a preoperative statin treatment. Preoperative characteristics, pathological results, and surgical details of patients are summarized in Table 1. Patients with and without preoperative statin treatment presented similar characteristics and intraoperative details, except for age and the incidence of pre-existing diabetes mellitus that were higher in the statin group. Postoperative outcomes of both groups are displayed in Table 2. No difference was found in terms of morbidity and length of hospital stay between both groups.

**Table 1:** Preoperative characteristics, pathological and surgical details of patients in the groups with and without preoperative statin treatment

	Statin treatment, N = 141	No statin treatment, N = 355	P-value
Age, years*	69 (65–76)	65 (56–74)	** < 0.001**
Sex (women)	58 (41)	178 (50)	0.070
Body-mass index, kg/m^2^*	25.5 (22.5–28.8)	24.9 (21.9–28.1)	0.830
Active smoker	34 (24)	72 (20)	0.348
Pre-existing diabetes	34 (24)	56 (16)	**0.030**
Jaundice	106 (75)	281 (79)	0.335
Preoperative biliary stenting	85 (60)	226 (64)	0.483
ASA score I-III	133 (94)	340 (96)	0.489
Highest CA 19–9, U/ml*	136 (26–491)	130 (31–388)	0.759
Tumor size on pathology, mm*	30 (25–40)	30 (23–37)	0.418
pT stage 1–2	29 (21)	60 (17)	0.337
pN0/pN1/pN2	38 (27)/53 (38) /50 (35)	73 (21)/163 (46)/119 (33)	0.169
Lymph node ratio*	0.120 (0–0.321)	0.125 (0.045–0.279)	0.685
Vascular invasion (V1)^a^	80 (57)	216 (61)	0.400
Tumor grade G1-G2	102 (72)	230 (65)	0.107
Classic PD^b^	52 (37)	128 (36)	0.863
PJ anastomosis	109 (77)	272 (77)	0.870
Operation time, min*	325 (268–389)	330 (269–397)	0.446
Intraoperative blood loss, ml*	400 (250–700)	500 (300–850)	0.090
Intraoperative blood transfusion	39 (28)	93 (26)	0.740
Vascular resection	20 (14)	76 (21)	0.066

Data appear as number and percentage

*ASA*: American Society of Anesthesiologists, *CA* carbohydrate antigen, *CT* computed tomography, *PD* pancreatoduodenectomy, *PJ* pancreaticojejunal

^*^Median and interquartile range

^a^Defined as microvascular invasion on pathology (V1 according to TNM staging)

^b^The rest of the patients underwent pylorus-preserving PD

Significant p-values appear in bold

**Table 2:** Postoperative outcomes of patients in the groups with and without preoperative statin treatment

	Statin treatment, N = 141	No statin treatment, N = 355	P-value
Overall complications	94 (67)	226 (64)	0.528
Minor complications	53 (38)	136 (38)	0.881
Major complications	41 (29)	90 (26)	0.396
CCI*	8.7 (0–29.6)	20.9 (0–27.6)	0.142
Delayed gastric emptying	29 (21)	89 (25)	0.288
Pancreatic fistula	10 (7)	34 (10)	0.380
Hemorrhage	14 (10)	34 (10)	0.905
Surgical-site infection	19 (13)	54 (15)	0.623
Length of stay, days*	14 (8–20)	14 (9–21)	0.209

Data appear as number and percentage

*CCI* Comprehensive Complication Index

^*^Median and interquartile range

### Survival and recurrence

**Fig. 1:**
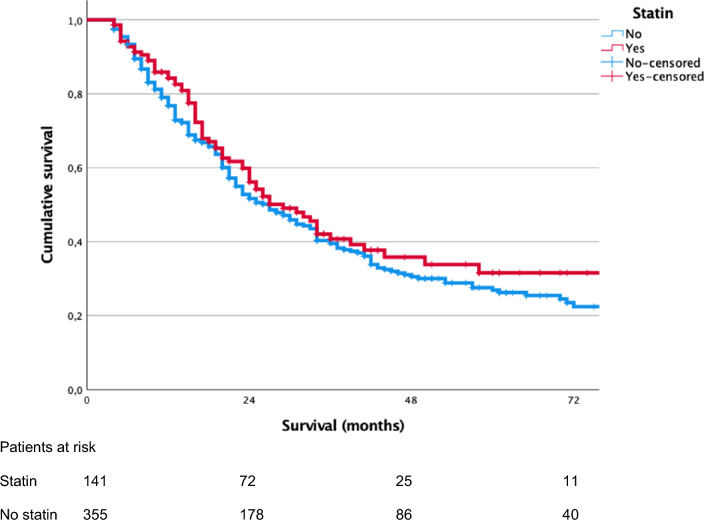
Kaplan–Meier curves of overall survival (OS) of patient with and without preoperative statin treatment (median OS: statin, 29 months, 95% CI 22–36 vs. no statin, 27 months, 95% CI 22–32, p = 0.370)

**Fig. 2:**
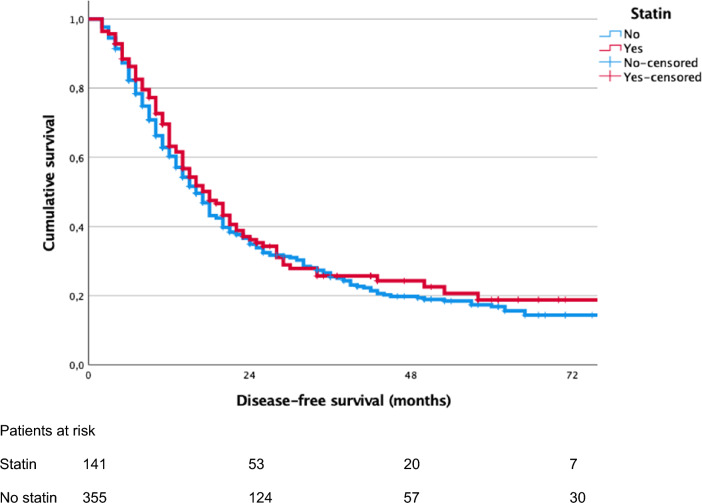
Kaplan–Meier curves of disease-free survival (DFS) of patient with and without preoperative statin treatment (median DFS: statin, 18 months, 95% CI 14–22 vs*.* no statin, 16 months, 95% CI 14–18, p = 0.430)

Median OS of patients with statin treatment was 29 months (95% CI 22–36) and 27 months (95% CI 22–32) for patients without preoperative statin treatment (p = 0.370). Median DFS were similar in the groups with and without statin treatment (18 months, 95% CI 14–22 vs*.* 16 months, 95% CI 14–18, p = 0.430). Kaplan–Meier curves comparing OS and DFS of patients with and without statin treatment are shown in Fig. 1 and Fig. 2. No gender-related median difference of OS between patients with and without statin treatment was found, respectively (women: 34 months, 95% CI 21–47 vs. 30 months, 95% CI 23–37, p = 0.406 and men: 26 months, 95% CI 17–35 vs. 24 months, 95% CI 19–29, p = 0.578). Within a median follow-up of 50 months (95% CI 45–55), the recurrence rates were 75/141 (53%) in the statin group and 200/355 (56%) in the group without statin treatment (p = 0.525). In the statin and non-statin groups, 75% (105/141) and 78% (277/355) of patients received postoperative chemotherapy, respectively (p = 0.395).

### Prognostic factors of survival

On univariable Cox regression, preoperative statin treatment was not found as a predictor of OS (HR 0.9, 95% CI 0.7–1.2, p = 0.376). On multivariable analysis, lymph node involvement (HR 1.9, 95% CI 1.6–2.2, p < 0.001), tumor differentiation (HR 1.3, 95% CI 1.1–1.6, p = 0.003), and postoperative chemotherapy (HR 0.5, 95% CI 0.4–0.7, p < 0.001) were identified as prognostic factors of OS. Table 3 summarizes the uni- and multivariable analyses for predictors of OS.

**Table 3:** Uni- and multivariable Cox regressions to determine prognostic factors of overall survival

	Univariable HR (95% CI)	P-value	Multivariable HR (95% CI)	P-value
Age		0.659		
≤ 60 years	Reference			
> 60 years	1.1 (0.8–1.4)	0.659		
Body-mass index				
≤ 25 kg/m^2^	Reference			
> 25 kg/m^2^	1.1 (0.8–1.3)	0.681		
Diabetes				
No	Reference			
Yes	1.3 (0.9–1.7)	0.113		
Smoking				
No	Reference			
Yes	0.9 (0.7–1.2)	0.446		
Biliary stenting				
No	Reference			
Yes	1.2 (1.0–1.6)	0.109		
ASA score				
I/II	Reference			
III/IV	1.2 (0.9–1.4)	0.153		
CA19-9				
≤ 42 U/ml	Reference			
> 42 U/ml	1.1 (0.8–1.5)	0.392		
Statin treatment				
No	Reference			
Yes	0.9 (0.7–1.2)	0.376		
T stage*				
T1–2	Reference			
T3–4	1.3 (1.0–1.8)	0.066	1.0 (0.8–1.4)	0.808
Lymph node involvement				
No	Reference			
Yes	1.8 (1.5–2.1)	** < 0.001**	1.9 (1.6–2.2)	** < 0.001**
Resection margin				
R0	Reference		Reference	
R1	1.4 (1.1–1.8)	**0.010**	1.3 (1.0–1.7)	0.053
Differentiation				
G1–2	Reference		Reference	
G3–4	1.4 (1.2–1.7)	** < 0.001**	1.3 (1.1–1.6)	**0.003**
Microvascular invasion				
No (V0)	Reference			
Yes (V1)	1.0 (0.8–1.3)	0.901		
Perineural invasion				
No (Pn0)	Reference			
Yes (Pn1)	0.8 (0.6–1.2)	0.293		
Adjuvant chemotherapy				
No	Reference		Reference	
Yes	0.8 (0.6–1.0)	0.095	0.5 (0.4–0.7)	** < 0.001**

*ASA* American Society of Anesthesiologists, *CA* carbohydrate antigen, *HR* hazard ratio

^*^Based on the 8th edition of the AJCC TNM classification for pancreatic cancer

Patients at risk

Significant p-values appear in bold

## Discussion

The present study assessing the impact of perioperative statin treatment on the long-term outcomes after surgery for PDAC did not find a difference in OS and DFS in this cohort. Independent predictors of OS were lymph node involvement, tumor differentiation, and adjuvant chemotherapy.

It has been hypothesized that statin had anti-cancer properties. Via the inhibition of HMG-CoA-reductase, statins block the transformation of HMG-CoA to geranyl-pyrophosphate and farnesyl-pyrophosphate (Di Bello et al. 2020). This blockage has an effect on decreasing not only cholesterol, but also inflammation, tissue remodeling, angiogenesis, and cell proliferation, as well as inducing apoptosis (Di Bello et al. 2020). The latter effects explain the potential anti-cancer properties of statins. Some studies have shown that patients with statin treatment had better survival in several types of solid cancers. Meta-analyses confirmed these findings for example in gastric and colorectal cancers (Cai et al. 2015; Yuan et al. 2022). In pancreas cancer, a 2019 systematic review and meta-analysis of 27 studies showed that statin users had a reduced risk of developing PDAC compared to patients without statin treatment (OR 0.70, 95% CI 0.60–0.82, p < 0.0001)(Archibugi et al. 2019). Moreover, a recent Norwegian study based on a nationwide registry found that patients under statin medication (n = 621) had better OS than non-statin users (n = 1993, HR: 0.87, 95% CI 76–97) (Støer et al. 2021). A 2018 meta-analysis found similar results with an improved survival in pancreatic cancer patients under statin treatment (HR: 0.75, 95% CI 0.59–0.90) (Jian-Yu et al. 2018). Significant heterogeneity between included studies was noticed. A 2020 meta-analysis even found that the positive effect on survival was more important among surgically resected pancreatic cancer patients (HR 0.5, 95% CI 0.32–0.76) (Tamburrino et al. 2020). It is important to bear in mind that observational studies might have selection and immortal-time biases, so the preceding findings need to be interpreted cautiously. A large study using the SEER-Medicare database (n = 17,372) and the inverse-probability weighting method to eliminate immortal-time bias showed that statin treatment initiated within 6 months after cancer diagnosis did not improve 3-year OS (Emilsson et al. 2018).

The present study assessed survival in patients who have been operated on, which is different from most of the observational studies on statins in cancer, where only non-operated patients are included, or a heterogeneous cohort of operated and non-operated patients is evaluated. In the studied specific cohort of PDAC patients operated upfront, it is interesting to hypothesize why no survival difference was found. First, it corroborates other studies on operated and non operated PDAC patients suggesting that statin treatment has no effect on long-term survival. For instance, a similar cross-sectional study by Tamburrino et al*.* including 430 patients who underwent pancreatic resection showed that DFS and disease-specific survival (DSS) were not influenced by a statin treatment (median DSS: statin, 37 vs. no statin, 34 months, p = 0.878)(Tamburrino et al. 2021). Of note, in the previously cited study, not all patients underwent pancreatoduodenectomy (69%) and 39% of the patients had neoadjuvant chemotherapy, which is different from the present study. The high rate of adjuvant chemotherapy after PDAC resection in this cohort (77%) might be another explanation, as adjuvant chemotherapy has been shown to improve OS of pancreatic cancer patients, such as in the present cohort. Other potential hypotheses are that the treatment duration and the therapeutic adherence to the statin treatment are important determinants. Unfortunately, these details were not available in this cohort. A final hypothesis pertains to the type of statin treatment. A meta-analysis by Tamburrino et al*.* found that rosuvastatin was associated with better outcomes in pancreas cancer patients, on the contrary of other statin types (Tamburrino et al. 2020). The present study included different statin subtypes without restriction.

In the present cohort, only patients who were operated upfront were included. The reason for not including patients who underwent neoadjuvant chemotherapy and for including only patients with PDAC of the pancreatic head was to have a cohort as homogeneous as possible. If such patients were included, it would have been necessary to take into account this supplementary element in the multivariable analysis or perform a subgroup analysis. Moreover, as neoadjuvant treatment was only recently implemented in the centers included in this study and various chemotherapy regimens were used, the number of patients with neoadjuvant would have been relatively small compared to the rest of the cohort and the heterogeneity more important.

Some limitations of this study should be mentioned. Data on the subtype of statin treatment (e.g., atorvastatine, simvastatine, others) were not available. Furthermore, the duration of the statin treatment was not known at time of data analysis. It was only known if patients had the statin treatment for more than a month prior to surgery. Another potential important information is the compliance to the statin treatment that was not available in the chart review. The retrospective design of the study has inherent limitations, such as errors during collection data or missing data.

This exploratory study suggests that using statin as an anti-cancer treatment for PDAC patients might not be useful in patients who undergo upfront surgery. Results might be different in a population of PDAC patients who benefit from neoadjuvant chemotherapy or in a patient cohort of borderline or locally advanced PDAC. This would be interesting to evaluate in future cohort or cross-sectional studies if statins impact survival in these subtypes of pancreatic cancer patients. Moreover, results of this study cannot be generalized to overall cancer patients, in particular patients who did not have surgery as upfront oncological treatment.

In summary, this international multicentric cross-sectional study did not identify preoperative statin treatment as a prognostic factor of survival in patients who underwent upfront surgery for PDAC. Factors predictive of OS were related to the biology of the tumor and adjuvant chemotherapy.

## Data Availability

The datasets generated and/or analyzed during the current study are available from the corresponding author on reasonable request.

## References

[CR1] Archibugi L, Arcidiacono PG, Capurso G (2019). Statin use is associated to a reduced risk of pancreatic cancer: a meta-analysis. Dig Liver Dis.

[CR2] Bassi C, Dervenis C, Butturini G (2005). Postoperative pancreatic fistula: an international study group (ISGPF) definition. Surgery.

[CR3] Cai H, Zhang G, Wang Z (2015). Relationship between the use of statins and patient survival in colorectal cancer: a systematic review and meta-analysis. PLoS ONE.

[CR4] Conroy T, Hammel P, Hebbar M (2018). FOLFIRINOX or gemcitabine as adjuvant therapy for pancreatic cancer. N Engl J Med.

[CR5] Di Bello E, Zwergel C, Mai A, Valente S (2020). The innovative potential of statins in cancer: new targets for new therapies. Front Chem.

[CR6] Dindo D, Demartines N, Clavien P-A (2004). Classification of surgical complications: a new proposal with evaluation in a cohort of 6336 patients and results of a survey. Ann Surg.

[CR7] Emilsson L, García-Albéniz X, Logan RW (2018). Examining bias in studies of statin treatment and survival in patients with cancer. JAMA Oncol.

[CR8] Fristrup C, Detlefsen S, Hansen CP, Ladekarl M (2016). Danish Pancreatic Cancer Database. Clin Epidemiol.

[CR9] Horan TC, Gaynes RP, Martone WJ (1992). CDC definitions of nosocomial surgical site infections, 1992: a modification of CDC definitions of surgical wound infections. Infect Control Hosp Epidemiol.

[CR10] Jian-Yu E, Graber JM, Lu S-E (2018). Effect of metformin and statin use on survival in pancreatic cancer patients: a systematic literature review and meta-analysis. Curr Med Chem.

[CR11] Shimagaki T, Sugimachi K, Mano Y (2023). A new scoring system with simple preoperative parameters as predictors of early recurrence of pancreatic ductal adenocarcinoma. PLoS ONE.

[CR12] Slankamenac K, Graf R, Barkun J (2013). The comprehensive complication index: a novel continuous scale to measure surgical morbidity. Ann Surg.

[CR13] Støer NC, Bouche G, Pantziarka P (2021). Use of non-cancer drugs and survival among patients with pancreatic adenocarcinoma: a nationwide registry-based study in Norway. Acta Oncol.

[CR14] Tamburrino D, Crippa S, Partelli S (2020). Statin use improves survival in patients with pancreatic ductal adenocarcinoma: a meta-analysis. Dig Liver Dis.

[CR15] Tamburrino D, Guarneri G, Pagnanelli M (2021). Chemopreventive agents after pancreatic resection for ductal adenocarcinoma: legend or scientific evidence?. Ann Surg Oncol.

[CR16] Wente MN, Bassi C, Dervenis C (2007). Delayed gastric emptying (DGE) after pancreatic surgery: a suggested definition by the International Study Group of Pancreatic Surgery (ISGPS). Surgery.

[CR17] Wente MN, Veit JA, Bassi C (2007). Postpancreatectomy hemorrhage (PPH): an International Study Group of Pancreatic Surgery (ISGPS) definition. Surgery.

[CR18] Yuan M, Han S, Jia Y (2022). Statins are associated with improved survival of patients with gastric cancer: a systematic review and meta-analysis. Int J Clin Pract.

